# Ursolic Acid Inhibits Superoxide Production in Activated Neutrophils and Attenuates Trauma-Hemorrhage Shock-Induced Organ Injury in Rats

**DOI:** 10.1371/journal.pone.0111365

**Published:** 2014-10-31

**Authors:** Tsong-Long Hwang, Hsin-I Shen, Fu-Chao Liu, Hsin-I Tsai, Yang-Chang Wu, Fang-Rong Chang, Huang-Ping Yu

**Affiliations:** 1 Graduate Institute of Natural Products, School of Traditional Medicine, College of Medicine, Chang Gung University, Taoyuan, Taiwan; 2 Chinese Herbal Medicine Research Team, Healthy Aging Research Center, Chang Gung University, Taoyuan, Taiwan; 3 Department of Anesthesiology, Chang Gung Memorial Hospital, Taoyuan, Taiwan; 4 College of Medicine, Chang Gung University, Taoyuan, Taiwan; 5 School of Pharmacy, College of Pharmacy, China Medical University, Taichung, Taiwan; 6 Chinese Medicine Research and Development Center, China Medical University Hospital, Taichung, Taiwan; 7 Graduate Institute of Natural Products, College of Pharmacy, Kaohsiung Medical University, Kaohsiung, Taiwan; Imperial College London, Chelsea & Westminster Hospital, United Kingdom

## Abstract

Neutrophil activation is associated with the development of organ injury after trauma–hemorrhagic shock. In the present study, ursolic acid inhibited the superoxide anion generation and elastase release in human neutrophils. Administration of ursolic acid attenuated trauma–hemorrhagic shock-induced hepatic and lung injuries in rats. In addition, administration of ursolic acid attenuated the hepatic malondialdehyde levels and reduced the plasma aspartate aminotransferase and alanine aminotransferase levels after trauma–hemorrhagic shock. In conclusion, ursolic acid, a bioactive natural compound, inhibits superoxide anion generation and elastase release in human neutrophils and ameliorates trauma–hemorrhagic shock-induced organ injury in rats.

## Introduction

Trauma-hemorrhagic shock is known to be followed by many life-threatening sequelae [1], [2]. Trauma-hemorrhagic shock induces the overproduction of proinflammatory mediators, which plays an important role in the process of organ injury [3]–[5]. Previous studies suggest that neutrophils are activated after trauma–hemorrhagic shock, and are essential in the development of organ dysfunction [6]–[8]. In this regards, inhibition of neutrophil function using drugs may be essential to treat trauma-hemorrhagic shock-induced organ dysfunction.

Neutrophil infiltration is associated with organ injury, including trauma-hemorrhagic shock [1]. Activated neutrophils are reported to produce cytotoxins, including the superoxide anion and proteases. In addition, oxidant stress leads to tissue destruction in various diseases [10]. The formation of superoxide anion in neutrophils is associated with organ damage [11], [12]. Neutrophil granules contain several stored proteases [12]. Elastase, a serine protease in chrymotrypsin family, has important role in the progress of various inflammatory diseases [13]–[15]. In this regards, it is essential to reduce neutrophil oxidative stress and elastase level following injury.

Ursolic acid, a natural pentacyclic triterpenoid carboxylic acid, is the antiinflammatory agent. The mechanisms of ursolic acid were evaluated in this study. Our results suggest that ursolic acid reduces the production of superoxide anion and release of elastase in human neutrophils. We also show that ursolic acid attenuates trauma-hemorrhagic shock-induced hepatic injury in rats.

## Materials and Methods

### 1. Preparation of Ursolic Acid

Ursolic acid, 3-beta-hydroxyurs-12-en-28-oic acid, was purchased from Santa Cruz Biotechnology (Santa Cruz, CA), and the purity is >98%. Ursolic acid was dissolved in dimethyl sulfoxide (DMSO; Sigma) and the total final concentration of DMSO did not exceed 0.4% in cell study.

### 2. Preparation of Neutrophils

Blood samples were drawn from healthy human donors (20–28 years old). The protocol was approved by the Institutional Review Board of Chang Gung Memorial Hospital. All participants were well informed and completed their written consent. This consent procedure was also approved by the Institutional Review Board of Chang Gung Memorial Hospital. Neutrophils were isolated with a standard method of dextran sedimentation before centrifugation in a Ficoll-Hypaque gradient and the hypotonic lysis of erythrocytes [16]. Purified neutrophils that contained >98% viable cells, as determined by the trypan blue exclusion method, were resuspended in Ca^2+^-free Hanks' balanced salt solution (HBSS) buffer at pH 7.4 and kept at 4°C before use.

### 3. Neutrophil Fractionation

Neutrophils were treated for 30 minutes with 1 mM PMSF and disrupted by sonication in relaxation buffer (100 mM KCl, 3 mM NaCl, 3.5 mM MgCl_2_, 1 mM ATP, 1 mM EGTA, and 10 mM Pipes; pH 7.3). The supernatant was centrifuged at 100000 g for 20 min. Unbroken cells were removed by centrifugation at 300 g for 5 min, and the supernatant was then centrifuged at 100000 g for 20 min at 4°C to produce the cytosolic and plasma membrane fractions. Superoxide anion was measured at 37°C after the addition of 160 µM NADPH to 800 µl of relaxation buffer containing 0.5 mg/ml ferricytochrome *c*, 4 × 10^6^ cell equivalents of membrane extract, 1.2×10^7^ cell equivalents of cytosol, 2 µM GTP-γ-S, and 100 µM sodium dodecyl-sulfate (SDS). Drugs were incubated for 2 min before NADPH oxidase assembly by SDS.

### 4. Assay of Superoxide Anion Production

The assay of the generation of superoxide anion was based on the superoxide dismutase (SOD)-inhibitable reduction of ferricytochrome *c* [16]. In brief, after supplementation with 0.5 mg/ml ferricytochrome *c* and 1 mM Ca^2+^, neutrophils (6×10^5^ cells/ml) were equilibrated at 37°C for 2 min and incubated with ursolic acid for 5 min. Cells were activated with 100 nM N-formyl-methionyl-leucyl-phenylalanine (FMLP) in the pre-priming with 1 µg/ml cytochalasin B (FMLP/CB) for 3 min. Changes in absorbance with the reduction of ferricytochrome *c* at 550 nm were continuously monitored with a double-beam, six-cell positioner spectrophotometer with constant stirring (Hitachi U-3010, Tokyo, Japan).

### 5. Assay of Elastase Release

The assay of the release of elastase was performed using MeO-Suc-Ala-Ala-Pro-Valp-nitroanilide as the elastase substrate [17], [18]. Briefly, after supplementation with MeO-Suc-Ala-Ala-Pro-Val-p-nitroanilide (100 µM), neutrophils (6×10^5^/ml) were equilibrated at 37°C for 2 min and incubated with ursolic acid for 5 min. Cells were activated by FMLP (100 nM) in the presence of CB (0.5 µg/ml), and changes in absorbance at 405 nm were continually monitored for 15 min. The results are expressed as a percentage of elastase release in the FMLP/CB-activated, drug-free control system.

### 6. Superoxide Anion-Scavenging Activity

The superoxide anion-scavenging ability of ursolic acid was determined using xanthine/xanthine oxidase (XO) in a cell-free system [19]. After 0.1 mM xanthine was added to the assay buffer (50 mM Tris, pH 7.4, 0.3 mM WST-1, and 0.02 U/ml XO) for 15 min at 30°C, the absorbance associated with the superoxide anion-mediated WST-1 reduction was measured at 450 nm.

### 7. Rats

Adult male Sprague-Dawley rats were used in this study. All animal experiments were performed according to the guidelines of the *Animal Welfare Act* and *The Guide for Care and Use of Laboratory Animals* from the National Institutes of Health. All procedures and protocols were approved by the Institutional Animal Care and Use Committee of Chang Gung Memorial Hospital. The rats were obtained from the National Science Council Experimental Animal Center.

### 8. Rat Trauma-Hemorrhagic Shock Model

A trauma-hemorrhagic shock rat model was used in the study [20]. Twenty-four male Sprague-Dawley rats were divided into 4 *groups* according to a table of *random numbers.* All rats were caged individually in the animal house with controlled humidity 70–75%, temperature (24–25°C) and lighting (light– dark cycle every 12 hours). The animals were provided with water and basal diet and at least 1 week was allowed for the animals to adapt to the environment. Animals were fasted overnight but allowed free water access before the experiment. Trauma-hemorrhagic shock and resuscitation was then performed as described previously [20]. In brief, rats were anesthetized with isoflurane inhalation, and soft tissue trauma was performed with midline laparotomy. The polyethylene catheters were placed in the right femoral vein and both femoral arteries. To reduce postoperative pain, the wounds were provided with 1% lidocaine. The rats were allowed to awaken, after which they were bled within 10 minutes to reach the mean arterial pressure of 35 to 40 mmHg. The mean arterial pressure was maintained between 35 to 40 mmHg for 90 minutes. Four times the volume of the shed blood with Ringer’s lactate were then resuscitated for 60 minutes. The rats received ursolic acid (1 mg/kg, intravenously) or the vehicle (about 0.2 ml DMSO) at 30 minutes from the beginning of the resuscitation period. Sham animals underwent all procedures; however, neither resuscitation nor hemorrhage was performed. Ursolic acid or vehicle was administered in sham rats. The rats were humanely sacrificed at 24 hours after sham operation or resuscitation.

### 9. Assay of Hepatic Injury

At 24 hours after trauma-hemorrhagic shock or sham operation, blood samples were drawn. Hepatic injury was measured with plasma aspartate aminotransferase (AST) and alanine aminotransferase (ALT) levels.

### 10. Assay of Malondialdehyde Levels

Malondialdehyde in the liver was measured as described previously [21]. Briefly, the malondialdehyde levels were analyzed with thiobarbituric acid assay and were measured by reading the absorbance at 535 nm.

### 11. Histological Examination of Lungs

For histological examination, tissues of lungs were fixed in 10% formalin in phosphate-buffered saline for 24 hours and were sent to the histology laboratory at Chang Gung University for further processing. Briefly, the sections were embedded in paraffin. These were then cut (4–5 µM) and mounted on glass slides. Sections of lungs were stained with hematoxylin-eosin, observed under the microscope (Nikon Eclipse TS100) at a magnification of ×400 for changes in morphology, and photographed using a camera (SPOT, RTcolor; Diagnostic Instrument, Iowa City, IA) attached to the microscope.

### 12. Statistical Analysis

Data are shown as the mean±SEM. Statistical analysis was performed using Student's *t* test or oneway analysis of variance followed by Tukey's multiple-comparison test. In all of the analysis, the value of *p*<0.05 was considered significant.

## Results

### 1. Ursolic Acid Reduced Superoxide Anion Generation and Elastase Release in Human Neutrophils

**Figure 1:**
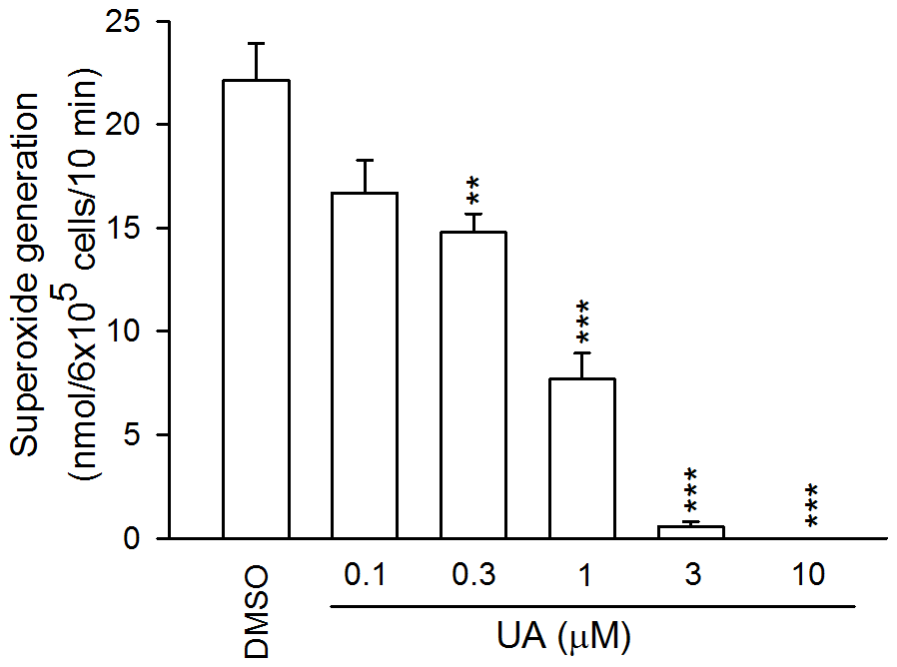
Effects of ursolic acid (UA) on superoxide anion generation in N-formyl-methionyl-leucyl-phenylalanine (FMLP) in the pre-priming with cytochalasin B (FMLP/CB)-activated human neutrophils. Data are shown as mean±SEM (n = 5). *p<0.05, **p<0.01, ***p<0.001 compared to the control.

**Figure 2:**
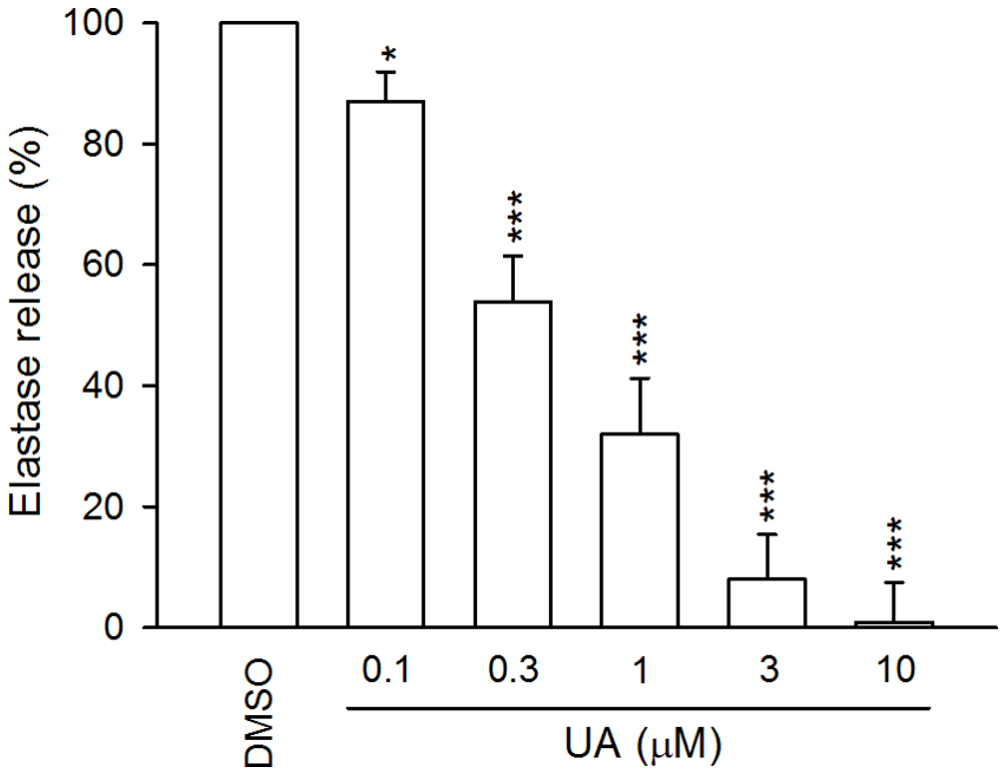
Effects of ursolic acid (UA) on elastase release in N-formyl-methionyl-leucyl-phenylalanine (FMLP) in the pre-priming with cytochalasin B (FMLP/CB)-activated human neutrophils. Data are shown as mean±SEM (n = 4). *p<0.05, **p<0.01, ***p<0.001 compared to the control.

Ursolic acid (0.3–10 µM) reduced superoxide anion release in FMLP/CB-activated cells in a concentration-dependent manner (Fig. 1). In addition, ursolic acid (0.1–10 µM) caused significant inhibition of the release of elastase (Fig. 2).

### 2. Ursolic Acid Failed to Change Superoxide Anion Generation in the Cell-Free Systems

**Figure 3:**
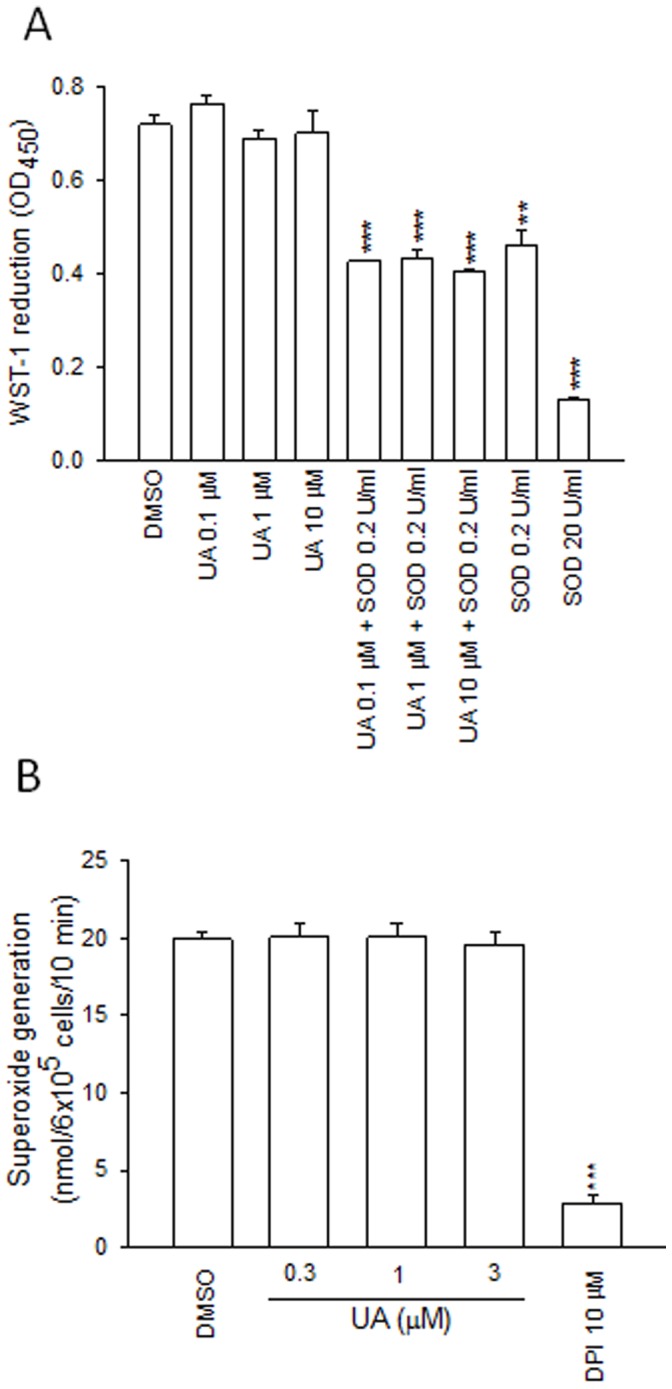
Effects of ursolic acid (UA) on superoxide anion production in a cell-free xanthine/xanthine oxidase system. (A) Reduction of WST-1 was determined in the presence of UA with or without superoxide dismutase (SOD). (B) A reactive mixture of the neutrophil cytosolic fraction and membrane fraction was preincubated with DMSO, UA (1, 3, and 10 µM), or diphenyleneiodonium (DPI). Data are shown as mean±SEM (n = 3 or 4). ***p<0.001 compared to the control.

Ursolic acid failed to change WST-1 reduction. Furthermore, ursolic acid did not influence the removal of superoxide anion by SOD (Fig. 3A). As shown in Fig. 3B, ursolic acid (1, 3, and 10 µM) did not alter the superoxide anion generation in isolated neutrophil membranes. Diphenyleneiodonium, a NADPH oxidase inhibitor, inhibited the production of superoxide anion. These data suggested that the inhibition of ursolic acid on the production of superoxide anion did not occur via the inhibitory effect of NADPH oxidase activity or the scavenging of superoxide anion.

### 3. Ursolic Acid Attenuated Trauma-Hemorrhagic Shock-Induced Hepatic and Lung Injuries in Rats

**Figure 4:**
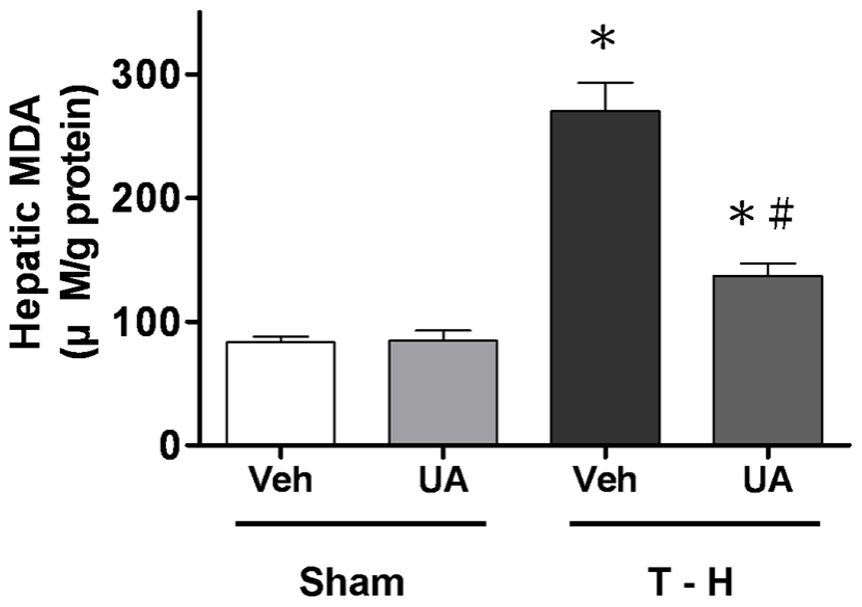
Effects of ursolic acid (UA) treatment on malondialdehyde (MDA) concentration in rats at 24 hr after sham operation (Sham) or trauma-hemorrhagic shock (T-H). Rats were administered with either vehicle (Veh) or UA. Data are expressed as mean±SEM of (n = 6). ^*^ *p*<0.05 compared to Sham; ^#^ *p*<0.05 compared to T–H+Veh.

**Figure 5:**
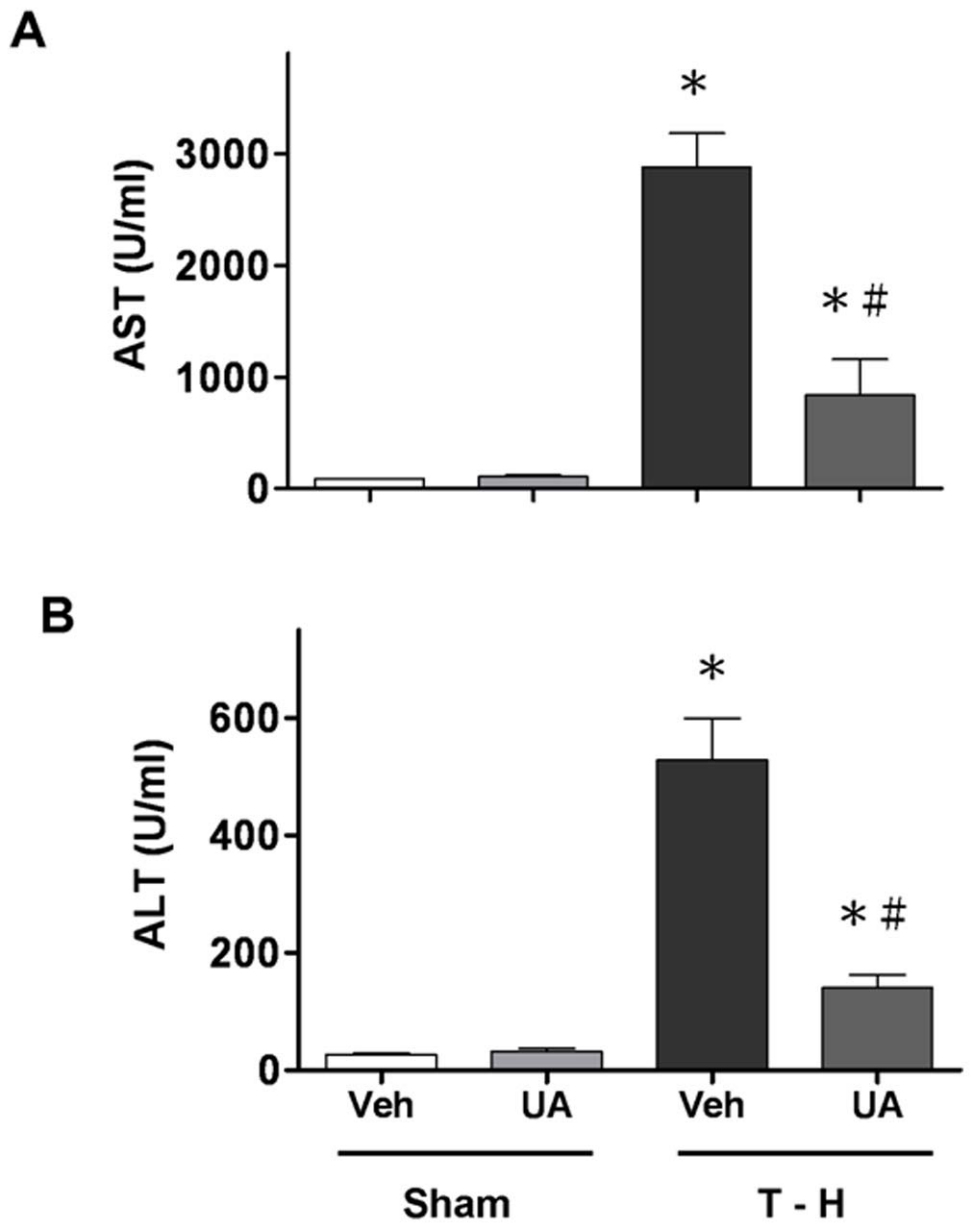
Effects of ursolic acid (UA) treatment on plasma (A) aspartate aminotransferase (AST) and (B) alanine aminotransferase (ALT) concentration in rats at 24 hr after sham operation (Sham) or trauma-hemorrhagic shock (T–H). Rats were administered with either vehicle (Veh) or UA. Data are shown as mean±SEM (n = 6). ^*^ *p*<0.05 compared to Sham; ^#^ *p*<0.05 compared to T–H+Veh.

**Figure 6:**
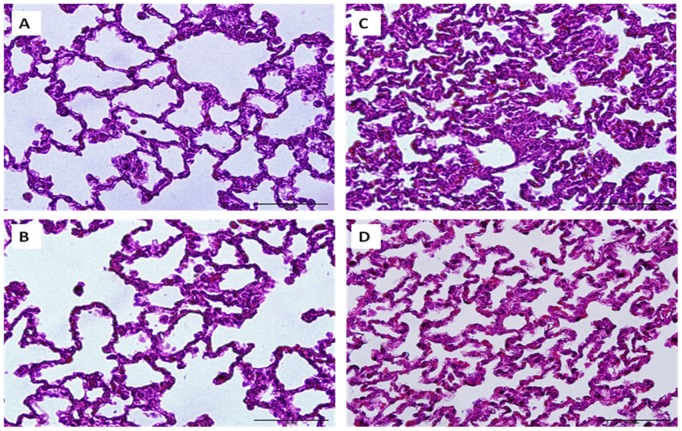
Histological effects of ursolic acid (UA) on lungs of rats after a sham operation (Sham) or trauma–hemorrhagic shock (T–H). Representative photomicrographs of lungs of (A) sham animals receiving vehicle, (B) sham animals receiving UA, (C) T–H animals receiving vehicle, and (D) T–H animals receiving UA. Tissue sections were stained with hematoxylin-eosin, examined at an original magnification of ×400, and photographed.

As shown in Fig. 4, malondialdehyde levels were increased significantly in the liver obtained from trauma-hemorrhagic shock animals compared to sham animals. In sham animals, ursolic acid did not change tissue malondialdehyde levels. Ursolic acid treatment ameliorated the increase in malondialdehyde levels in the liver (Fig. 4). In addition, the plasma AST and ALT levels obtained from trauma-hemorrhagic shock animals were increased significantly compared with those from sham animals (Fig. 5). Administration of ursolic acid attenuated the increased liver injury; nonetheless, these parameters remained higher compared with sham rats (Fig. 5). Representative photomicrographs of lungs are presented for sham animals treated with vehicle (Fig. 6A), sham animals treated with ursolic acid (Fig. 6B), trauma-hemorrhagic shock animals treated with vehicle (Fig. 6C), and trauma-hemorrhagic shock animals treated with ursolic acid (Fig. 6D). Administration of ursolic acid attenuated trauma-hemorrhagic shock-induced lung injury.

## Discussion

Inappropriate activation of neutrophils may contribute to tissue damage. Previous studies suggest that the infiltration of neutrophils is associated with trauma-hemorrhagic shock-induced organ dysfunction [6]–[8]. In the present study, ursolic acid showed inhibitory effects on superoxide anion generation and elastase release in human neutrophils. The results also suggested that ursolic acid treatment attenuated trauma-hemorrhagic shock-induced hepatic injury in rats.

Previous studies have indicated that enhanced oxidative stress and proteases level are related to the pathogenesis of inflammatory diseases [9], [13]. The production of superoxide anion and release of elastase in neutrophils are important inflammatory responses which lead to damage of surrounding tissues [10], [13]. Ursolic acid potently suppressed the production of superoxide anion and release of elastase in activated human neutrophils. These results suggest that ursolic acid can act as an antiinflammatory agent.

The production of superoxide anion activated in human neutrophils can be reduced by regulation of cellular signaling pathways, and also by scavenging radical. The formation of superoxide anion was not inhibited by ursolic acid in cell-free xanthine/xanthine oxidase system, suggesting that ursolic acid does not inhibit the release of superoxide anion through scavenging superoxide anion production. Furthermore, ursolic acid did not show direct inhibition of NADPH oxidase. These results suggest that ursolic acid has its inhibitory effect upstream of NADPH oxidase. Obviously, further studies are required to identify the molecular target and action of mechanism of ursolic aicd in human neutrophils.

In the present study, the beneficial effects of ursolic acid in trauma-hemorrhagic shock-induced hepatic injury were examined. Our results suggested that administration of ursolic acid decreased hepatic malondialdehyde levels and attenuated trauma-hemorrhagic shock-induced hepatic and lung injuries. Though the precise mechanism of ursolic acid administration in reducing trauma-hemorrhagic shock-induced organ injury remains unclear, this study may provide evidence that inhibition of superoxide anion generation and elastase release serves as the potential mechanism in decreasing organ injury after trauma-hemorrhagic shock.

## Conclusions

In conclusion, these results suggest that ursolic acid attenuates superoxide anion generation and elastase release in activated human neutrophils. In addition, our results suggest that ursolic acid may have future potential as a novel adjunct for improving depressed organ function after adverse circulatory conditions.
